# Supplementary material for: Modulation of electrical activity of proteinoid microspheres with chondroitin sulfate clusters
Source: PLoS One. 2024 Dec 4;19(12):e0313077. doi: 10.1371/journal.pone.0313077 (PMC11616837; doi:10.1371/journal.pone.0313077)
Supplement: Attachment — Submitted filename: Response to Reviewers.pdf [file pone.0313077.s001.pdf]

PONE-D-24-32280

Modulation of electrical activity of proteinoid microspheres with chondroitin sulfate clusters

PLOS ONE

Dear Dr. Mougkogiannis,

Thank you for submitting your manuscript to PLOS ONE. After careful consideration, we feel that it has merit but does not fully meet PLOS ONE's publication criteria as it currently stands. Therefore, we invite you to submit a revised version of the manuscript that addresses the points raised during the review process.

Please submit your revised manuscript by Nov 25 2024 11:59PM. If you will need more time than this to complete your revisions, please reply to this message or contact the journal office at plosone@plos.org. When you're ready to submit your revision, log on to https://www.editorialmanager.com/pone/ and select the 'Submissions Needing Revision' folder to locate your manuscript file.

Please include the following items when submitting your revised manuscript:

- A rebuttal letter that responds to each point raised by the academic editor and reviewer(s). You should upload this letter as a separate file labeled 'Response to Reviewers'.
- A marked-up copy of your manuscript that highlights changes made to the original version. You should upload this as a separate file labeled 'Revised Manuscript with Track Changes'.
- An unmarked version of your revised paper without tracked changes. You should upload this as a separate file labeled 'Manuscript'.

If you would like to make changes to your financial disclosure, please include your updated statement in your cover letter. Guidelines for resubmitting your figure files are available below the reviewer comments at the end of this letter.

If applicable, we recommend that you deposit your laboratory protocols in protocols.io to enhance the reproducibility of your results. Protocols.io assigns your protocol its own identifier (DOI) so that it can be cited independently in the future. For instructions see: https://journals.plos.org/plosone/s/submission-guidelines#loc-laboratory-protocols. Additionally, PLOS ONE offers an option for publishing peer-reviewed Lab Protocol articles, which describe protocols hosted on protocols.io. Read more information on sharing protocols at https://plos.org/protocols?utm_medium=editorial-email&utm_source=authorletters&utm_campaign=protocols.

We look forward to receiving your revised manuscript.

Kind regards,

Lei Zhang
Academic Editor
PLOS ONE

Journal requirements:

When submitting your revision, we need you to address these additional requirements.

1. Please ensure that your manuscript meets PLOS ONE's style requirements, including those for file naming. The PLOS ONE style templates can be found at

https://journals.plos.org/plosone/s/file?id=wjVg/PLOSOne_formatting_sample_main_body.pdf
and

https://journals.plos.org/plosone/s/file?id=ba62/PLOSOne_formatting_sample_title_authors_affiliations.pdf

2. Thank you for stating the following financial disclosure: [The research was supported by EPSRC Grant EP/W010887/1 ``Computing with proteinoids'']. Please state what role the funders took in the study. If the funders had no role, please state: "The funders had no role in study design, data collection and analysis, decision to publish, or preparation of the manuscript." If this statement is not correct you must amend it as needed. Please include this amended Role of Funder statement in your cover letter; we will change the online submission form on your behalf.

3. Please review your reference list to ensure that it is complete and correct. If you have cited papers that have been retracted, please include the rationale for doing so in the manuscript text, or remove these references and replace them with relevant current references. Any changes to the reference list should be mentioned in the rebuttal letter that accompanies your revised manuscript. If you need to cite a retracted article, indicate the article's retracted status in the References list and also include a citation and full reference for the retraction notice.

Additional Editor Comments (if provided):

[Note: HTML markup is below. Please do not edit.]

Reviewers' comments:

Reviewer's Responses to Questions

Comments to the Author

1. Is the manuscript technically sound, and do the data support the conclusions?

The manuscript must describe a technically sound piece of scientific research with data that supports the conclusions. Experiments must have been conducted rigorously, with appropriate controls, replication, and sample sizes. The conclusions must be drawn appropriately based on the data presented.

Reviewer #1: Yes

Reviewer #2: Yes

---

2. Has the statistical analysis been performed appropriately and rigorously?

Reviewer #1: Yes

Reviewer #2: Yes

---

3. Have the authors made all data underlying the findings in their manuscript fully available?

The PLOS Data policy requires authors to make all data underlying the findings described in their manuscript fully available without restriction, with rare exception (please refer to the Data Availability Statement in the manuscript PDF file). The data should be provided as part of the manuscript or its supporting information, or deposited to a public repository. For example, in addition to summary statistics, the data points behind means, medians and variance measures should be available. If there are restrictions on publicly sharing data—e.g. participant privacy or use of data from a third party—those must be specified.

Reviewer #1: Yes

Reviewer #2: Yes

---

4. Is the manuscript presented in an intelligible fashion and written in standard English?

PLOS ONE does not copyedit accepted manuscripts, so the language in submitted articles must be clear, correct, and unambiguous. Any typographical or grammatical errors should be corrected at revision, so please note any specific errors here.

Reviewer #1: Yes

Reviewer #2: Yes

---

5. Review Comments to the Author

Please use the space provided to explain your answers to the questions above. You may also include additional comments for the author, including concerns about dual publication, research ethics, or publication ethics. (Please upload your review as an attachment if it exceeds 20,000 characters)

Reviewer #1: This paper investigates the modulation of the electrical activity of proteinoid microspheres via interaction with chondroitin sulfate (CS) clusters. The authors aim to explore how this hybrid system, combining proteinoids—synthetic, neuron-like structures—with components of the brain's extracellular matrix (CS), could mimic aspects of synaptic plasticity and learning. The findings suggest that CS clusters significantly influence the electrical behavior of proteinoid microspheres, especially in terms of spiking dynamics, offering new insights into molecular mechanisms underlying learning and information processing. The study stands out as an innovative attempt to combine biomimetic structures with complex biochemical networks, with potential applications in neuromorphic computing and unconventional bio-inspired systems.

What makes this work novel is its attempt to merge synthetic proteinoids with natural biomolecules like CS, creating a hybrid system that could provide clues about the origins of neuron-like communication. This reminds me of research on memristive nanoparticles, where complex interactions at nanoscale interfaces lead to adaptive electrical behavior, suggesting parallels in the system's potential to store and process information.

Given the adaptive and spike-driven nature of this hybrid system, have you considered exploring its suitability for reservoir computing? The spontaneous and externally modulated spiking behavior seems to be a promising candidate for such computational architectures.

Since the model they use for this type of analysis is essentially an integrate and fire model, I wonder if they could say anything about reservoir computing with this type of systems and how the random network can affect the information processing.

As far as I know, they only work that does something like this is this one: https://arxiv.org/abs/2407.20547, or this https://www.nature.com/articles/s44335-024-00002-4 although they do not call it RC.

Authors:

We appreciate the suggestion to consider reservoir computing for our hybrid proteinoid microsphere system. It was insightful. Indeed, the adaptive and spike-driven nature of our system does share some similarities with reservoir computing architectures using spiking neural networks, as highlighted in recent work by Karki et al. and Stoffel and Tandale.

Text added:

‘’Reservoir computing with spiking neural networks shows promise. It can model chaotic dynamics and forecast time series. Reservoir computing systems can analyze complex, time-dependent data. This is due to the stochastic network architecture and intrinsic temporal dynamics of spiking neurons~\cite{karki2024neuromorphic,stoffel2024spiking,fortulan2024achieving}. Our proteinoid microsphere networks have spontaneous, regulated electrical activity. They may have similar computing powers. The random arrangement of microspheres and the unstable links from chondroitin sulfate clusters may act as a physical reservoir. The spiking activity of individual microspheres modifies incoming signals in a nonlinear way. This study focuses on the electrical properties of proteinoid microsphere networks. Future research may explore their use as novel computing devices. This may mean studying how different network designs affect processing. Also, it may include looking at the effects of external modulations. Our proteinoid system's computation may outperform neuron models in reservoir computing. This could reveal insights into both biological and artificial information processing.’’

I also have questions about this type of nanoparticles. The system reminds me of the type of nanoparticles studied by Simon Brown. The system is different. See for instance: https://www.science.org/doi/10.1126/sciadv.aaw8438. I wonder whether randomness will play a role. There are not many studies about this, except for those with memristive systems. I don't think the model they use is memristive, but maybe some parallels could be drawn. Analytically, it would be hard to do anything without using techniques from disordered systems (for memristive devices, which I know a bit better, examples are these: https://onlinelibrary.wiley.com/doi/10.1002/andp.202300090, https://www.science.org/doi/10.1126/sciadv.abh1542), but for SNN maybe the standard papers are those by John Hertz and collaborator. Of course, I am not asking to do these calculations, but directions that theorists and experimentalists can investigate along the line of density, percolation transitions, average connectivity (Etc). See for instance https://arxiv.org/pdf/1606.04466 or https://arxiv.org/html/2409.00412v1.

I think this paper is very nice, I enjoyed reading it quite a lot.

Authors:

Thank you for sharing those additional references and your comments; we have added the following paragraph to the discussion.

‘’Our research on proteinoid microspheres is similar to other self-organising nanoscale systems. This includes the nanoparticle networks studied by Brown and others~\cite{doi:10.1126/sciadv.aaw8438}. Both show emergent, collective behaviors from interactions among individual components. Although the principles vary, they are the same at the core level. The influence of randomness and disorder is probably considerable in our

system, although it is not clearly represented in our current methodology. Future research may explore if ideas from disordered systems, informed by studies on memristive networks, could improve our understanding of the dynamics in proteinoid microsphere networks~\cite{https://doi.org/10.1002/andp.202300090,doi:10.1126/sciadv.abh1542}. For instance, examining how network characteristics like as density, average connectedness, and percolation thresholds affect the system's electrical behaviour may provide significant insights~\cite{stolzenburg2016neural,meshulam2024statistical}. Also, using methods from studies on disordered brain networks~\cite{meshulam2024statistical} could give new ways to understand our system. Though outside this study, these directions offer promise. They may guide research to clarify the principles behind proteinoid microsphere networks and similar self-organizing nanoscale systems''

Minor corrections:

- Clarify the dosage impact on electrical activity, particularly how variability in electrical potential changes across CS concentrations.

In order to assess the impact of dosage on electrical activity, we have added a new figure. See below.

Figure \ref{fig:variability} shows a non-linear correlation. The mean electrical potential is related to CS concentration. The minimum potential was -3.91 V at 1.86 mg/ml. The maximum was 1.70 V at 1.10 mg/ml. The standard deviation of electrical potential significantly diminishes with elevated CS concentration. This shows that high CS concentrations stabilize the electrical properties of the proteinoid solutions. The water control (0 mg/ml CS) exhibited the minimal fluctuation, establishing a baseline for comparison.

Figure caption : \bf Concentration-dependent effects of chondroitin sulfate on electrical potential in proteinoid solutions} \textcolor{red} {The graph shows the link between chondroitin sulphate (CS) concentration and the average electrical potential of proteinoid solution. Data points denote mean potentials (red circles) and standard deviations (error bars) for each tested CS concentration (1.10, 1.86, 4.00, and 10.20 mg/ml), plus a water control (0 mg/ml). The plot shows a non-linear correlation between CS concentration and electrical potential. It is most variable at moderate concentrations (1.86 mg/ml). The electrical potential fluctuates less with higher CS concentrations. This suggests a stabilising effect at high concentrations. The water control demonstrates minimal variability, serving as a baseline for comparison. This visualisation shows a complex link between CS concentration and electrical traits in proteinoid solutions. It highlights CS's potential role in regulating and stabilising electrical activity in these systems.}

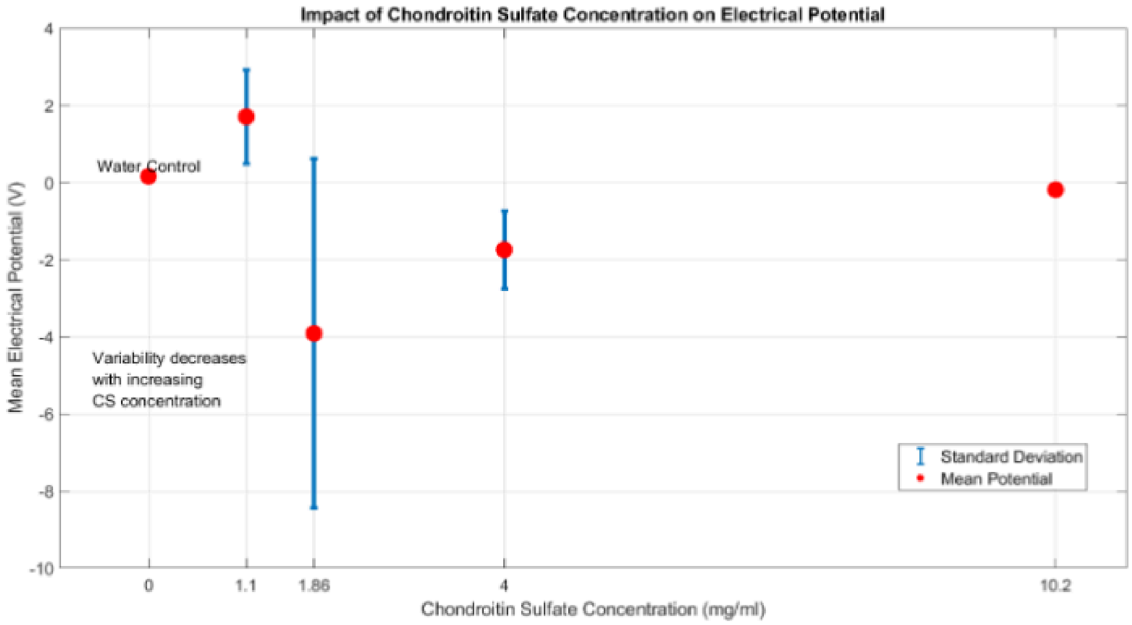

Fig 4. Concentration-dependent effects of chondroitin sulfate on electrical potential in proteinoid solutions The graph shows the link between chondroitin sulphate (CS) concentration and the average electrical potential of proteinoid solution. Data points denote mean potentials (red circles) and standard deviations (error bars) for each tested CS concentration (1.10, 1.86, 4.00, and 10.20 mg/ml), plus a water control (0 mg/ml). The plot shows a non-linear correlation between CS concentration and electrical potential. It is most variable at moderate concentrations (1.86 mg/ml). The electrical potential fluctuates less with higher CS concentrations. This suggests a stabilising effect at high concentrations. The water control demonstrates minimal variability, serving as a baseline for comparison. This visualisation shows a complex link between CS concentration and electrical traits in proteinoid solutions. It highlights CS's potential role in regulating and stabilising electrical activity in these systems.

- Add more details on the statistical methods used for analyzing spike data.

The analysis of spike data used a thorough statistical method. It found the amplitude and period distributions of the recorded electrical activity. For each distribution, we computed key stats: mean, median, standard deviation, and quartiles. This showed the data's central tendency and spread. To clarify the distributions' traits, we calculated their skewness and kurtosis. Skewness measures a distribution's asymmetry. We computed it using the given equation:

\begin{equation}

\text{Skewness} = \frac{\mathbb{E}[(X-\mu)^3]}{\sigma^3}

\end{equation}

where X is the random variable, μ is the mean, σ is the standard deviation, and $\mathbb{E}$ denotes the expected value. Kurtosis, which quantifies the tailedness of the distribution, was computed using:

\begin{equation}

\text{Kurtosis} = \frac{\mathbb{E}[(X-\mu)^4]}{\sigma^4}

\end{equation}

These metrics provide key insights into the amplitude and period distributions. They help us better interpret the spiking behaviour. We applied a gamma distribution to the amplitude data. This characterized its probability. We used maximum likelihood estimation to find the shape and rate parameters.

- The transition between experimental results and theoretical modeling could benefit from more explanatory context.

In this section, we investigated the signal processing capabilities of proteinoid-chondroitin mixtures (1.8 mg/ml) using a thalamocortical network model based on Izhikevich neurons.

In this section, we investigated the signal processing capabilities of proteinoid-chondroitin mixtures (1.8 mg/ml) using a thalamocortical network model based on Izhikevich neurons. We used theoretical modeling. It links our findings on electrical activity in proteinoid-chondroitin sulfate complexes to their potential neural-like signaling functions. Our experiments show, especially in the spiking behaviour, that these complexes have dynamics like neural signalling. This resemblance led us to investigate if these systems could mimic complex neural network behaviours. We chose a thalamocortical network model for our analysis for several reasons. The thalamocortical circuit is key to mammalian brains. It helps with sensory processing, attention, and awareness. Secondly, this circuit has a mix of dynamics. It has oscillatory patterns and complex signal transmission. These match the varied spiking behaviour in our proteinoid-chondroitin sulphate complexes. The Izhikevich neurone model is efficient and can replicate various firing patterns. It is the best choice for simulating these dynamics while keeping biological plausibility.

Reviewer #2: Mougkogiannis et al. worked on the interaction between proteinoid microspheres and chondroitin sulfate (CS) clusters, as well as how this interaction regulates the electrical activity of the proteinoid microspheres. The findings hold significance for understanding the application of biomaterials in neural system simulation, especially regarding the comprehension of molecular mechanisms underlying synaptic plasticity. The authors have a clear idea throughout the article. The pictures are very intuitive and interesting. The manuscript may be

further improved by considering following points:

1. Introduction:

Previous studies have reported that proteinoid microspheres can exhibit electrical spikes. Therefore, it could more clearly highlight the innovative aspects of this study, such as the experimental methods, theoretical models, or new mechanisms expected to be discovered.

Prior research showed that proteinoid microspheres can produce electrical spikes~\cite{mougkogiannis2024proto,mougkogiannis2024spike,mougkogiannis2024transducing,muller1994stability}. Our study greatly enhances this knowledge using various novel methods. We use advanced experimental methods, like high-precision electrochemical techniques and multi-parametric imaging. They provide a detailed view of the electrical activity in these microspheres. Secondly, we present a new framework. It integrates molecular mechanics optimization, solvation analysis, and modified Hodgkin-Huxley models. This method models the complex interactions at a molecular level. It yields new insights into the mechanisms that govern the electrical properties. Our research is the first to study how chondroitin sulphate concentration affects the electrical properties of proteinoid microspheres. It uncovers a new aspect of their function. We use the Izhikevich neuron model in a new way. It mimics thalamocortical dynamics in these systems. It connects proteinoid behaviour and neuronal signaling. These developments may reveal new ways to process signals and create ``memories'' in proto-cells. They could change our understanding of the origins of neural-like behaviour. This may lead to bio-inspired computer systems.

2. Results:

- There are discrepancies between the numbering of some figures and their references within the manuscript. For example, Figure 2 is mentioned but does not appear to correspond to the described content

Thank you for bringing this to our attention. The discrepancy in Figure references has been resolved.

-The abbreviation of chondroitin sulfate has been written as CS, so do not write the full name in the following text.

Thank you for pointing that out. The abbreviation "CS" has be used for chondroitin sulfate in the text, and unnecessary definitions have been removed.

3. Discussion:

-The discussion section still reads more like a restatement of the research findings rather than a comprehensive summary and reflection on the content of the study. Moreover, the limitations of the current research have not been adequately addressed. It would be beneficial to expand on these points.
-Appropriate relevant literature can be cited to discuss the potential applications in a broader biological or medical context. A recent article about CS promoting tissue repair should provide the needed references https://doi.org/10.1016/j.carbpol.2023.120738. I also recommend the authors read and cite the following review about how electrical signals synergize with matrix cells to regulate neural functions: https://doi.org/ 10.1002/advs.202003390.

Our results on CS and proteinoid microspheres match recent studies on CS's role in tissue healing and regeneration. Hao et al. found that CS helps tissue repair~\cite{HAO2023120738}. It does this by boosting cell growth, movement, and development. It also regulates inflammation and modulates the extracellular matrix. CS-modulated proteinoid microspheres have electrical activity. It may help repair processes, especially in brain tissue. CS can affect the electrical properties of proteinoid structures. This suggests possible uses in neural tissue engineering and regenerative medicine. Future research may explore how CS's electrical regulation affects tissue repair and regeneration, especially in the nervous system. Also, our work improves understanding of how electrical impulses and extracellular matrix components interact to regulate brain activity. Wan et al. studied the interaction between bone and nerves in the skeleton. It emphasized the effect of electrical signals from the bones on nerve growth and function~\cite{wan2021crosstalk}. The electrical activity reported in proteinoid-CS complexes may resemble elements of bone-nerve communication. This analogy suggests our proteinoid-CS system may be a simpler model. It can help examine the complex links among the extracellular matrix, electrical signaling, and central nervous system (CNS) function. Future research may explore how the electrical traits of proteinoid-CS complexes affect neural cells. It could reveal new insights into nerve cell growth and repair. It may also help in treating neurological diseases.

4. Reference:
Please ensure the reference format complies with the journal's requirements.

---

6. PLOS authors have the option to publish the peer review history of their article (what does this mean?). If published, this will include your full peer review and any attached files.

If you choose “no”, your identity will remain anonymous but your review may still be made public.

Do you want your identity to be public for this peer review? For information about this choice, including consent withdrawal, please see our Privacy Policy.

Reviewer #1: No

Reviewer #2: No
